# Development and Potential Usefulness of the COVID-19 Ag Respi-Strip Diagnostic Assay in a Pandemic Context

**DOI:** 10.3389/fmed.2020.00225

**Published:** 2020-05-08

**Authors:** Pascal Mertens, Nathalie De Vos, Delphine Martiny, Christian Jassoy, Ali Mirazimi, Lize Cuypers, Sigi Van den Wijngaert, Vanessa Monteil, Pierrette Melin, Karolien Stoffels, Nicolas Yin, Davide Mileto, Sabrina Delaunoy, Henri Magein, Katrien Lagrou, Justine Bouzet, Gabriela Serrano, Magali Wautier, Thierry Leclipteux, Marc Van Ranst, Olivier Vandenberg, LHUB-ULB SARS-CoV-2 Working Diagnostic Group, Béatrice Gulbis

**Affiliations:** Author Affiliations: Department of Clinical Chemistry, LHUB-ULB, Université Libre de Bruxelles, Brussels, Belgium; Administrative and Management Unit, LHUB-ULB, Université Libre de Bruxelles, Brussels, Belgium; Quality Insurance Unit, LHUB-ULB, Université Libre de Bruxelles, Brussels, Belgium; Administrative and Management Unit, LHUB-ULB, Université Libre de Bruxelles, Brussels, Belgium; Department of Haematology, LHUB-ULB, Université Libre de Bruxelles, Brussels, Belgium; Department of Immunology, LHUB-ULB, Université Libre de Bruxelles, Brussels, Belgium; Department of Clinical Chemistry, LHUB-ULB, Université Libre de Bruxelles, Brussels, Belgium; Administrative and Management Unit, LHUB-ULB, Université Libre de Bruxelles, Brussels, Belgium; Department of Microbiology, LHUB-ULB, Université Libre de Bruxelles, Brussels, Belgium; Administrative and Management Unit, LHUB-ULB, Université Libre de Bruxelles, Brussels, Belgium; Administrative and Management Unit, LHUB-ULB, Université Libre de Bruxelles, Brussels, Belgium).; ^1^Coris BioConcept, Gembloux, Belgium; ^2^Department of Clinical Chemistry, LHUB-ULB, Université Libre de Bruxelles, Brussels, Belgium; ^3^Department of Microbiology, LHUB-ULB, Université Libre de Bruxelles, Brussels, Belgium; ^4^Medical Faculty and University Clinics, Institute for Virology, University of Leipzig, Leipzig, Germany; ^5^Division of Clinical Microbiology, Department of Laboratory Medicine, Karolinska Institutet, Stockholm, Sweden; ^6^Department of Microbiology, Immunology and Transplantation, KU Leuven, Leuven, Belgium; ^7^Clinical Department of Laboratory Medicine and National Reference Center for Respiratory Pathogens, UZ Leuven, Leuven, Belgium; ^8^Department of Clinical Microbiology, Centre Hospitalier Universitaire Sart-Tilman, Université de Liège, Liège, Belgium; ^9^Laboratory of Clinical Microbiology, Virology and Bioemergency, ASST Fatebene fratelli Sacco, Luigi Sacco Hospital, Milan, Italy; ^10^Innovation and Business Development Unit, LHUB-ULB, Université Libre de Bruxelles, Brussels, Belgium; ^11^Division of Infection and Immunity, Faculty of Medical Sciences, University College London, London, United Kingdom

**Keywords:** COVID-19, SARS-CoV-2, diagnostic, immunochromatographic test, antigen

## Abstract

**Introduction:** COVID-19 Ag Respi-Strip, an immunochromatographic (ICT) assay for the rapid detection of SARS-CoV-2 antigen on nasopharyngeal specimen, has been developed to identify positive COVID-19 patients allowing prompt clinical and quarantine decisions. In this original research article, we describe the conception, the analytical and clinical performances as well as the risk management of implementing the COVID-19 Ag Respi-Strip in a diagnostic decision algorithm.

**Materials and Methods:** Development of the COVID-19 Ag Respi-Strip resulted in a ready-to-use ICT assay based on a membrane technology with colloidal gold nanoparticles using monoclonal antibodies directed against the SARS-CoV and SARS-CoV-2 highly conserved nucleoprotein antigen. Four hundred observations were recorded for the analytical performance study and thirty tests were analyzed for the cross-reactivity study. The clinical performance study was performed in a retrospective multi-centric evaluation on aliquots of 328 nasopharyngeal samples. COVID-19 Ag Respi-Strip results were compared with qRT-PCR as golden standard for COVID-19 diagnostics.

**Results:** In the analytical performance study, the reproducibility showed a between-observer disagreement of 1.7%, a robustness of 98%, an overall satisfying user friendliness and no cross-reactivity with other virus-infected nasopharyngeal samples. In the clinical performance study performed in three different clinical laboratories during the ascendant phase of the epidemiological curve, we found an overall sensitivity and specificity of 57.6 and 99.5%, respectively with an accuracy of 82.6%. The cut-off of the ICT was found at CT <22. User-friendliness analysis and risk management assessment through Ishikawa diagram demonstrate that COVID-19 Ag Respi-Strip may be implemented in clinical laboratories according to biosafety recommendations.

**Conclusion:** The COVID-19 Ag Respi-Strip represents a promising rapid SARS-CoV-2 antigen assay for the first-line diagnosis of COVID-19 in 15 min at the peak of the pandemic. Its role in the proposed diagnostic algorithm is complementary to the currently-used molecular techniques.

## Introduction

Severe acute respiratory syndrome coronavirus 2 (SARS-CoV-2) constitutes a major health threat to humankind (1). In the absence of a vaccine and specific antiviral treatment, the containment of the pandemic relies mainly on the rapid identification and isolation of COVID-19 patients (2). In addition to chest computed tomography (CT-scan), this strategy is based on the availability of real-time reverse transcription polymerase chain reaction (qRT-PCR) to be performed on any suspect patient presenting specific symptoms (3). These symptoms being similar to those of the seasonal flu, it is currently not possible to test all patients with flu-like symptoms due to the lack of resources and available diagnostic tests. As mentioned in the audio interview of the New England Journal of Medicine on the 19th of March 2020 (2), the importance of establishing the correct diagnosis is central to giving the appropriate care to COVID-19 patients.

So far, several molecular-based tests have been developed and are being implemented in laboratories and reference centres with capabilities to perform such tests (see https://www.who.int/emergencies/diseases/novel-coronavirus-2019/technical-guidance/laboratory-guidance for details). However, the availability of molecular diagnostic tests is a concern as we face a worldwide shortage of the reagents. Although molecular diagnosis is the most sensitive and specific diagnostic method, the need for material, reagents and trained personnel limits the number of assays that can be performed and saturates the laboratories. Moreover, qRT-PCR still does not have a very rapid turnaround time (TAT).

The development of rapid diagnostic assays allows faster confirmation of a clinical suspicion of COVID-19, leading to earlier isolation and appropriate clinical care for patients with positive results. Several serological tests have been developed, but serological antibody-detection assays do not fulfill the requirement of the detection early after infection as the average incubation period of 3 to 5 days is too short for the development of an immune response (4).

From this perspective, Coris BioConcept (a Belgian manufacturer) has developed an immunochromatographic test (ICT) for the rapid detection of the SARS-CoV-2 antigen on nasopharyngeal specimens in ~15 min. Thanks to the results from previous research on SARS-CoV, the nucleoprotein was identified as the best target for a sensitive diagnostic sandwich assay using monoclonal antibodies (5–7). All monoclonal antibodies were initially generated using full-length SARS nucleocapsid protein (NP) and were subsequently tested with SARS-CoV-2 NP. The NP sequence for generating the antibody is reference AY291315.1 in Genbank (cfr. Supplementary Table 1). The SARS-CoV-2 shares a high similarity with bat coronaviruses, and the known SARS-CoV of the 2002–2003 epidemic (8) provided the opportunity to use previously developed reagents for developing a rapid diagnostic assay able to also detect the new SARS-CoV-2. The diagnostic technique consists of an anti-SARS-CoV capture antibody fixed onto a nitrocellulose strip and a labeled anti-SARS-CoV antibody migrating with the buffer and the sample.

Regarding the COVID-19 pandemic and the urgency of sharing relevant data, in this original research article we describe the analytical performance of the COVID-19 Ag Respi-Strip according to the requirements of the current European Directive 98/79/EC (9), the future European Regulation 2017/746 on *in vitro* diagnostic (IVD) medical devices (10), the Scandinavian SKUP-protocol (11) used for the validation of qualitative tests and the clinical performance obtained with a multi-centric retrospective study. In addition, we reflect on the risk management and the conditions to be fulfilled before implementation as a point-of-care test (POCT) outside the hospital.

## Materials and Methods

### Development of the COVID-19 Ag Respi-Strip

#### Antibodies and Antigen

Eleven antibodies (designed A to K) (12) were coated at various concentrations on nitrocellulose (Advanced Microdevices, India) with antibodies A to J coupled to colloidal gold beads (NanoQ, Belgium). Recombinant SARS-CoV nucleoprotein (recNP) preparation was obtained as described previously (12) and was coated on a nitrocellulose membrane or conjugated on colloidal gold nanoparticles. Recombinant his-tagged SARS-CoV-2 NP (recNP-2) has been produced in insect cells and purified (2-step purification), with a final purity > 90% (Genscript, Leiden, NL).

#### COVID-19 Ag Respi-Strip

The ICT strip consists of nitrocellulose laminated on a plastic backing, with colloidal-gold conjugates being dried on a conjugate pad (Ahlstrom-Munksjö, France) overlapping the bottom of the nitrocellulose. For preliminary direct detection, SARS-CoV and SARS-CoV-2 NPs were coated at 100 μg/mL, and gold-labeled antibodies were deposited at 0.85 μl/mm at 3 OD530 nm. The mean diameter of the gold nanoparticles is 40 nm. For the COVID-19 Ag Respi-Strip test, monoclonal antibodies directed against the SARS-CoV and SARS-CoV-2 highly conserved nucleoprotein antigen are coated on the nitrocellulose. Another monoclonal antibody is conjugated to colloidal gold nanoparticles (mAb-gold nanoparticle). The conjugate is immobilized on the conjugate pad. The volume of the mAb-gold nanoparticle used in the conjugate release pad is 3.36 μl per test (0.84 μl per mm, while the strips are 4 mm wide). During the development, tests analyzing the antibody reactivity and intensity were performed using serial dilutions of SARS-CoV-2 in a final volume of 300 μl of buffer (data not shown here; cfr. Supplementary Tables 2, 3). The results were determined after 15 min. For the experiments during development, the buffer volume was 300 μl, while the buffer volume for the final test has been set at 200 μl. During analytical and clinical performance studies, the final test used 200 μl of buffer volume.

**Figure 1:**
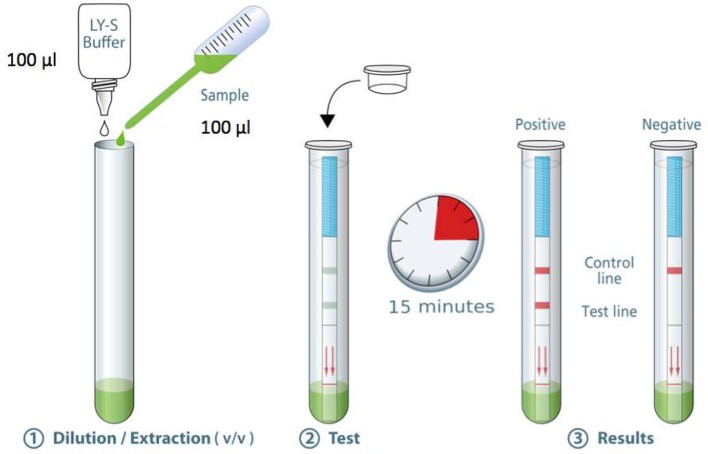
Standard operating procedure SARS-CoV-2 Respi-Strip® from Coris BioConcept.

The standard operating procedure for the COVID-19 Ag Respi-Strip is as follows (Figure 1): Transfer 100 μL of a nasopharyngeal sample [nasopharyngeal aspirates (NPA), nasopharyngeal washes or nasal/nasopharyngeal swabs (NPS)] in the collection tube. Add 100 μL of the LY-S dilution buffer to reach a dilution ratio of 1:2. The LY-S dilution buffer consists of TRIS buffer containing EDTA, NaN3 (<0.1%), a detergent and blocking agents. Cap the tube with the stopper. Stir thoroughly to homogenize the solution. Open the tube. Immerse the strip in the direction indicated and close the tube with the stopper. Allow to react for 15 min and read the result. Regarding interpreting the results, for a negative test result, a reddish-purple line appears at the Control line (C) position (upper line). No other band is present. For a positive test result, in addition to a reddish-purple band at the (C), a visible reddish-purple band appears at the Test line (T) position. The intensity of (T) may vary according to the quantity of antigens found in the sample. Any reddish purple line (T), even weak, should be considered as a positive result. An invalid test result is when the absence of a Control line indicates a failure in the test procedure. Repeat invalid tests with a new strip. Discard the closed tube according to biohazard rules (i.e., using personal protective equipment (PPE) including gloves, a medical a mask, goggles or a face shield, gown and physical containment such the Biological Safety Cabinet Class II to close the tube before discarding in a container for hazardous medical/biological waste). For correct judgement on how a weak color should be considered as a positive result, the Supplementary Table 4 shows pictures of real strips with strongly positive, weakly positive and negative samples.

#### ELISA

A F-bottom high binding 96-well microtiter plate (Greiner Bio-One GmbH) was coated with 50 μL recNP and recombinant maltose binding protein (MBP, 1 μg/mL) and incubated overnight at 4°C. The plate was washed with water and washing buffer (phosphate-buffered saline/0.5% Tween-20, PBS-T), and 200 μL blocking solution (PBS-T/5% milk powder) was added for 20 min. The blocking solution was discarded, and mAbs were added at 100 pg/mL in 50-μL blocking solution to recNP and MBP and incubated for 1 h. The plate was washed again, and 50 μL rabbit anti-mouse IgG/HRP (Dako) was added at a 1:1000 dilution for another 1 h incubation. The plate was washed, and 50 microliter TMB substrate solution was added. All incubations took place at room temperature. After 5–10 min, the enzymatic reaction was stopped by the addition of 50 μL 1 N H_2_SO_4_, and light absorption was measured with a photometer at 450 nm using 570 nm as a reference wavelength. Measurements were taken in duplicates. To obtain the final OD, the OD obtained with the control protein MBP was subtracted from the OD obtained with recNP.

#### Virus

SARS-CoV-2 passage 3 (SARS-CoV-2-Iso_01-Human-2020-02-07-Swe, accession no/GenBank no. MT093571) was cultured on Vero E6 cells. The titer was determined using a plaque assay, as described above, with a fixation of cells at 72 hpi. All experiments involving isolates of SARS-CoV-2 were performed at the Biosafety Level 3 Laboratory at the Public Health Agency of Sweden (Folkhälsomyndigheten, Stockholm, Sweden).

#### qRT-PCR

Samples were extracted using Direct-zol RNA MiniPrep kit (Zymo Research). qRT-PCR was run using E-gene SARS-CoV-2 primers/probe following World Health Organization advice (13).

### Analytical Performance Study

During the development phase, the analytical performance of the assay was performed using a 2-fold serial dilution of the virus (in viral culture medium) in parallel with titrating the same virus preparation on Vero E6 cells (by plaque assay) and testing by qRT-PCR.

For the analytical sensitivity and specificity obtained in a clinical biology lab setting, 60 samples from UZ Leuven, the National Reference Centre for the diagnosis of COVID-19 in Belgium, were analyzed in the laboratory LHUB-ULB (Laboratoire Hospitalier Universitaire Bruxelles—Universitair Laboratorium Brussel), Brussels. All samples were NPS in a viral transport medium (3 mL UTM). The analysis protocol with the COVID-19 Ag Respi-Strip was as follows: Of the 20 positive patient samples with a cycle threshold (CT) below 25, 10 of them were analyzed in duplicate; of the 20 weakly positive samples with CTs between 25 and 37.7, all were analyzed in duplicate; and of the 20 negative patient samples, 10 of them were analyzed in duplicate. Duplicate specimens were randomly chosen. For these 100 analyses, 4 observers delivered a qualitative result, resulting in 400 observations. The diagnostic efficacy of the COVID-19 Ag Respi-Strip was evaluated comparing the results with those previously obtained on fresh nasopharyngeal samples tested for SARS-CoV-2 with the reference qRT-PCR test (13, slightly adapted in NRC). Regarding the qRT-PCR reference test, total nucleic acid was extracted using NucliSens extraction on easyMAG (bioMérieux, Lyon, France), followed by the addition of a Phocine Distemper Virus (PDV) internal control (IC) (14). PCR amplification was performed on QuantStudio Dx (Thermo Fisher Scientific) using slightly adapted E-gene primers and a probe (13). After the run, the amplification plots were analyzed and interpreted using QuantStudio Test Development Software (version 1.0, Thermo Fisher Scientific).

ICT assays were performed following the manufacturer's instructions using high containment measures (Biological Safety Cabinet Class II). With these samples, the analytical performance study consisted of analytical sensitivity, analytical specificity, reproducibility (between-observer disagreement with 4 observers simultaneously reading the result) and robustness.

For the cross-reactivity study, experiments to assess the reactivity of the COVID-19 Ag Respi-Strip to other pathogens were conducted in the LHUB-ULB depending on specimen availability (*N* = 30, consisting of 20 NPA and 10 sputa). Clinical residual anonymized respiratory samples from patients with non-SARS-CoV-2 infections were tested. The concentrations of the pathogens fluctuated owing to the available stock, and the clinical specimens with the highest virus load were selected.

The user-friendliness study was performed according to the Scandinavian protocol SKUP/2004/35^*^ with 5 questioned operators responding independently to a checklist (11).

### Clinical Performance Study

To consider the implementation of the COVID-19 Ag Respi-Strip into the national diagnostic algorithm, an urgent multi-centric retrospective study aiming to assess the clinical performance of this rapid assay against current molecular methods (golden standard) was performed. Overall, 328 nasopharyngeal samples from symptomatic patients suspected of SARS-CoV-2 infections attending from 19th to 30th March 2020 in three university laboratories located in Belgium were tested following the manufacturer's instructions to assess the clinical sensitivity, clinical specificity, positive predictive value (PPV), negative predictive value (NPV) and accuracy in order to propose a diagnostic algorithm adapted to the current situation. This retrospective multi-centric evaluation integrates 322 randomly selected NPS [flocked swab + UTM 3 mL (or 1 mL of Amies) (Copan, Brescia, Italy)], 4 NPA (diluted with 3 mL of viral transport medium composed of veal infusion broth (Difco, Becton Dickinson, Sparks, MD, USA) supplemented with bovine albumin [Sigma Aldrich, St Louis, MO, USA)] (15) and 2 Broncho-Alveolar Lavage (BAL) of the biobanks at LHUB-ULB, UZ Leuven and CHU Liège. Aliquots of these patient samples were analyzed with COVID-19 Ag Respi-Strips and compared to the qRT-PCR result.

At the LHUB-ULB laboratory, viral RNA extraction was performed by the QIAsymphony DSP Virus/Pathogen Kit (QIAGEN), which extracted a 400-μl sample eluted in a 60-μl elution buffer for the Mini Kit and an 800-μl sample eluted in a 110-μl buffer for the Midi Kit, or by m2000 Sample Preparation SystemDNA Kit (Abbott) using a 1,000-μl manually lysed sample (700-μl sample + 800-μl lysis buffer from kit) eluted in a 90-μl elution buffer. A qRT-PCR IC was added at each extraction. qRT-PCR was performed using 10-μl of the extracted sample in the RealStar® SARS-CoV-2 RT-PCR Kit from Altona Diagnostics with a cut-off set at 40 CT.

The RT-PCR protocol used in Liege for the comparison was as follows: RNA was extracted from clinical samples (300 μL) on a Maxwell 48 device using the Maxwell RSC Viral TNA kit (Promega). Reverse transcription and RT-PCR were performed on a LC480 thermocycler (Roche) based on Charité's protocol for the detection of RdRp and E genes (16) using the Taqman Fast Virus 1-Step Master Mix (Thermo Fisher).

For UZ Leuven, a second qRT-PCR method was performed on a Panther Fusion (PF, Hologic, San Diego, USA) Open AccessTM SARS-CoV analysis. The analytical protocol was as follows: 500 μL UTM from the nasopharyngeal sample is added to a PF lysis tube, mixed by pipetting and loaded on the instrument. All following steps, including total nucleic acid extraction, reverse transcription and real-time PCR, are automatized on the instrument and were defined in the LDT-protocol using the myAccess software.

This SARS-CoV assay targets the following 2 SARS-CoV genes: the E-gene, for which primers and probers are slightly adapted from Corman et al. (13), and the gene ORF1-b. Amplification plots are analyzed by the system software using parameters defined in the LDT-protocol. A linear regression line y = 0.9993x + 5.4341 was constructed to normalize the difference in CT values found with the two methods used in UZ Leuven (y = Panther Fusion and x = Quantstudio). To obtain a cut-off, all positive qRT-PCR results were grouped per category of one CT and matched with the most frequent qualitative interpretation obtained with COVID-19 Ag Respi-Strip. At least 50% of the qualitative interpretations are found to be positive at the cut-off.

The study was approved by the ethical boards—P2020/191 for Hôpital Erasme, CE2020/65 for CHU Brugmann, AK/10-06-41/3907 for CHU Saint-Pierre and S63896 for UZ Leuven. For CHU of Liege, no specific approval was requested by the EC as a leaflet including the following statement is given to all admitted patients: “*According to the law of the 19th December 2008, any left-over of biological material collected from patients for their standard medical management and normally destroyed when all diagnostic analysis have been performed, can be used for validation of methods. The law authorizes such use except if the patient expressed an opposition when still alive (presume consent)*”.

### Risk Management

The risks and bottlenecks of the use of the COVID-19 Ag Respi-Strip will be presented in an Ishikawa diagram (17).

## Results

### Selection and Characterization of Monoclonal Antibodies

Four different assays were performed to assess the reactivity of antibodies toward SARS-CoV NP (I-III) and SARS-CoV-2 (IV) nucleocapsid proteins. Antibodies were tested using an ELISA on immobilized recNP showing various reactivities (Supplementary Table 2, column I). Prior to the use of antibodies in a sandwich detection assay, the antibodies were individually tested for their ability to capture and/or detect the target recNP in ICT format. The antibodies were thus coated onto nitrocellulose and tested using the recNP coupled to colloidal gold beads; reactivity was recorded as based on the visual intensity on the ICT strips (Supplementary Table 2, column II). Lastly, antibodies were coupled to colloidal gold beads and migrated on an ICT strip where the recNP was immobilized; results are recorded in Supplementary Table 2, column III. As can be observed, the reactivity of antibodies in the ELISA assay does not predict their ability to work properly as capture or detection reagents in an ICT format, as described previously for other targets (12). Moreover, antibodies with no detectable activity, such as detection reagents (antibodies B, C, D, column III), show weak to good capture capability (Supplementary Table 2, column II).

Comparing the reactivity on both recNP and recNP-2, the overall reactivity was higher on recNP-2 although antibodies were initially elicited and selected against recNP. This may reflect different protein preparation protocols. More interestingly, antibodies B, C, and H, which were reacting against recNP, did not react with recNP-2 at all, leading to the hypothesis that these antibodies may react with an epitope which is specific for recNP and not present on recNP-2.

Sandwich ICT assays were performed by combining antibodies working as capture reagents (coated on nitrocellulose) and antibodies working as detection reagents (gold-labeled). These ICTs were tested using recNP at a similar concentration for all tests (100 ng/mL). All 20 combinations giving a visible signal were assessed on their ability to detect the SARS-CoV-2 virus (Supplementary Table 3).

The assay on the virus was performed using a serial dilution of culture supernatant. The relative intensities observed on recNP and on SARS-CoV-2 were similar, with Prototypes 4 and 5 giving the highest signals. The final COVID-19 Ag Respi-Strip, corresponding to Prototype 5 (i.e., the combination of antibodies A and J, as reported in Supplementary Table 3), was further characterized for analytical performance during the development of the IVD medical device.

### Analytical Performance Study

During the development phase, the analytical performance was first performed on 2-fold serial dilutions of recNP-2. The limit of detection (LOD) was defined as the last dilution that tested positive (15 tests, 2 independent readers). The assay was shown to have a detection level down to 250 pg/mL. The assay was tested on a supernatant of Vero E6 cells infected with SARS-CoV-2 virus. We have found that the assay can detect 5 × 10e3 pfu/mL, corresponding to a CT value of 23.7 in a qRT-PCR assay (Supplementary Table 5).

During validation in the clinical biology lab, all 130 strips used in this analytical study were valid with the exception of two. The analytical sensitivity for patients having a high viral load (CT < 25) was 74.2%, with an analytical specificity of 100.0% (Table 1). The reproducibility showed a between-observer disagreement of 1.7 % (*N* = 7/398). The robustness is 98.0%, showing only two out of 100 tests being invalid as they lacked the visual test control line because migration did not succeed. In all of the succeeded tests (N = 98/98), the reaction was fulfilled in time, that is, after 15 min.

**Table 1:** Analytical performance study.

	**UZ Leuven specimen**		**LHUB-ULB test**		**New COVID-19 Ag Respi-Strip (Saline Buffer)**
	**Number of samples**	**Lab**	**Observers**	**N observations**	**Results**
		**Interpretation (qRT-PCR)**			
**Positive samples (with qRT-PCR)**					
CT < 25	30	Positive	4	120	89/120 (74.2%)
25 < CT < 37.7	40	Weak positive	4	160	20/160 (12.5%)
**Negative samples**	30	Negative	4	120	120/120 (100.0%)

No cross-reactivity nor interference has been found in nasopharyngeal samples containing the following pathogens (overall *N* = 20): Coronavirus HKU1 (*N* = 2), Adenovirus (*N* = 3), Enterovirus (*N* = 2), Influenza A virus (*N* = 3), Influenza B virus (*N* = 3), human Metapneumovirus (*N* = 2), Parainfluenza virus (*N* = 1), Rhinovirus (*N* = 4), RSV (*N* = 2), and *Mycoplasma pneumoniae* (*N* = 1).

Because of the unavailability of nasopharyngeal samples with *Staphylococcus aureus*, the cross-reactivity study could not be properly performed, and the test circumstances were simulated in approximation with *N* = 10 sputa with abundant cultures of *S. aureus*. The cross-reactivity for *S. aureus* in sputum, which is not the prescribed sample type for COVID-19 diagnostics and moreover has unknown matrix interference, gave one false positive test result. On the contrary, in all weakly and strongly positive SARS-CoV-2 observations with the nasopharyngeal samples of UZ Leuven, no false positive results have been detected with the COVID-19 Ag Respi-Strip (*N* = 0/109).

Cross-reactivity was also checked for viruses from culture supernatants: Coronaviruses OC43, NL63, 229E, and HKU1 as well as SARS-CoV. No cross-reactivity was observed for the four seasonal coronaviruses; cross-reactivity was observed for SARS-CoV, as expected.

The user-friendliness is satisfactory for the information in the instruction for use (IFU) of the manufacturer and for the time factors related to the pre-analytical and analytical phases. As the internal and external quality controls unrelated to the kit are not yet available, the overall rating for quality control is less satisfactory although the interpretation of the quality control line integrated in the strip is satisfactory even if some control lines showed a weak intensity. The user-friendliness evaluation related to the operation showed a very satisfactory rating (Supplementary Table 6).

### Clinical Performance Study

The overall clinical sensitivity is 57.6%, and the clinical specificity is 99.5%, with a PPV of 98.7%, an NPV of 77.7% and an accuracy of 82.6% (Table 2). Even if the overall sensitivity of 57.6% will detect 6 out of 10 random people with COVID-like symptoms presenting at the hospital, in the subpopulation of the most contagious patients with the highest viral load (i.e., with CT < 25), this test will detect 7 out of 10 positive COVID-19 patients. In the COVID-19 population, the CTs ranged from 9.4 to 39.4, and the cut-off of the COVID-19 Ag Respi-Strip was found at CT < 22. Below the cut-off, the clinical sensitivity was 95.0%. The study population showed that the majority of the symptomatic COVID-19 patients presented with high viral load (CT mean = 22.2). A small number of samples have been studied from symptomatic healthcare workers (*N* = 53), and the clinical performance showed a sensitivity of 68.0% and a specificity of 100.0% in this subpopulation.

**Table 2:** Clinical performance study.

	**LHUB-ULB**	**CHU Liège**	**UZ Leuven**	**OVERALL**
	***N* = 99**	***N* = 132**	***N* = 97**	***N* = 328**
**Prevalence**	30.3%	55.3%	29.9%	40.2%
CT: Mean	20.5	23.3	20.9	22.2
CT: Median	19.5	23.6	22.5	22.4
**Sensitivity**				
Overall	60.0%	60.3%	48.3%	57.6%
For CT < 25	85.7% (on *N* = 21)	76.7% (on *N* = 43)	58.3% (on *N* = 24)	73.9% (on *N* = 88)
**Specificity**	100.0%	98.3	100.0%	99.5%
**PPV**	100.0%	97.8%	100.0%	98.7%
**NPV**	85.2%	66.7%	81.9%	77.7%
**Accuracy**	87.9%	77.3%	84.5%	82.6%
Subpopulation of healthcare workers		*N* = 23	*N* = 30	*N* = 53
**Prevalence**		56.5%	40.0%	47.2%
CT: Mean		26.4	18.1	22.4
CT: Median		29.6	15.7	22.0
**Sensitivity**				
Overall		61.5%	75.0%	68.0%
For CT < 25		80.0% (on *N* = 5)	100.0% (on *N* = 9)	92.9% (on *N* = 14)
**Specificity**		100.0%	100.0%	100.0%
**PPV**		100.0%	100.0%	100.0%
**NPV**		66.7%	85.7%	77.8%
**Accuracy**		78.3%	90.0%	84.9%

**Figure 2:**
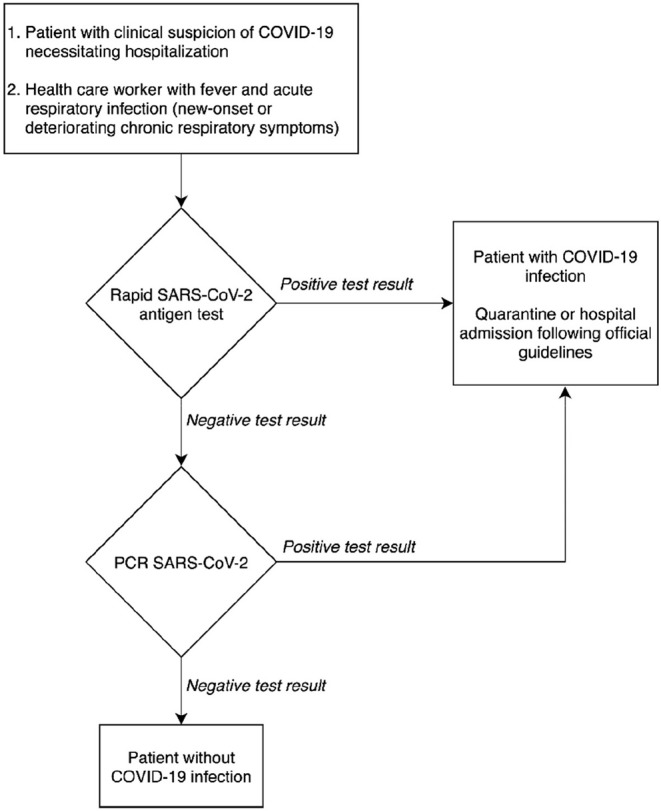
Proposal for a diagnostic decision algorithm.

Based on the results of the clinical performance study, a diagnostic decision algorithm is proposed in Figure 2.

### Risk Management

**Figure 3:**
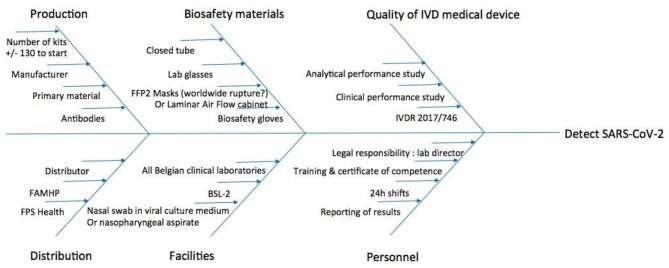
Ishikawa diagram of rapid SARS-CoV-2 diagnostic tests for Clinical Labs' implementation. BSL-2, Biosafety Level 2; FAMHP, Belgian Federal Agency for Medicines and Health Products; FFP2, Filtering Facepiece Respirator Class 2; FPS Health, Belgian Federal Public Service Health, Food Chain Safety and Environment; IVDR, European Regulation 2017/746 on *in vitro* Diagnostic Medical Devices.

To visualize the risks and bottlenecks of the use of the COVID-19 Ag Respi-Strip, an Ishikawa diagram has been created considering continuous risk management (Figure 3). The facilities of a clinical biology laboratory are recommended in the Ishikawa diagram to respond to the highest quality assurance and to permit lab technicians to handle the sample according to biosafety recommendations.

## Discussion

The extraordinary spread of SARS-CoV-2 has resulted in the need for an accelerated development of rapid and accurate laboratory diagnostic tests, allowing fast and accurate detection of infected patients (18). In this perspective, fast near-patient testing, or POCT, could represent an effective diagnostic tool for prompt clinical and quarantine decisions. Besides the recent certification of molecular POCT, such as Xpert®Xpress SARS-CoV-2 on GeneXpert (Cepheid) or ID NOW COVID-19 on Alere-i (Abbott), antigenic tests could represent a valuable alternative especially in the context of the worldwide shortage of reagents and instruments (which has already been reported for these molecular POCTs).

This research report is, according to our knowledge, the first to describe the analytical and clinical performance of a non-fluorescent ICT that can detect the SARS-CoV-2 antigen in nasopharyngeal samples. The antigen detection was already reported in a pre-peer-reviewed article but using a fluorescence ICT (19).

An analytical performance study was followed by a clinical performance study, both set up according to the requirements of European legislation.

With the analytical sensitivity of 74.2% found in a subpopulation with high SARS-CoV-2 viral load (CT < 25), the analytical specificity of 100.0% and robustness of 98.0%, the COVID-19 Ag Respi-Strip is a promising new technique. In addition, our multi-centric retrospective study confirms the promising results, with an overall clinical sensitivity of 57.6% and a clinical specificity of 99.5%. The clinical sensitivity is 68.0% in a subpopulation of healthcare workers and increases to 73.9% in a subpopulation of the most contagious patients with high viral load (CT < 25). In this subpopulation with high viral shedding (*N* = 88 with CT < 25), the clinical sensitivity was 73.9%. Looking at all patients in the clinical performance study, the majority of the positive test results had a high viral load: 66.7% of all positive test results had a CT value below 25. Shi et al. mention that the severe respiratory symptomatic stage is associated with high viral load (4).

Like many of the commercially available lateral flow assays, COVID-19 Ag Respi-Strip lacks sensitivity compared to currently used amplification based assays, such as qRT-PCR, and will most likely miss around four patients out of 10 that potentially might have COVID-19. However, additional clinical validation using dry swab samples directly immersed into the assay buffer might increase the sensitivity of the assay somewhat. The same setting (i.e., dry swab in LY-S buffer) will be required to assess the sensitivity of the assay if used as a POCT.

When we reflect on the cross-reactivity study, the number of samples analyzed for *S. aureus* was not sufficient, and sputum was not prescribed as a sample type in the manufacturer's IFU. It is not clear whether the false positive sputum (*N* = 1/10) from the cystic fibrosis patient was due to a matrix effect, its high-viscosity or the presence of *S. aureus*. Taking these factors into account, sputa from cystic fibrosis patients with higher viscosity and multi-pathogenic presence might lead to an unacceptable level of false positivity. Whether cystic fibrosis patients should be excluded from analysis with the COVID-19 Ag Respi-Strip and directly receive a diagnosis with qRT-PCR is not clear but could be judicious as long as we lack further evidence. Overall, at this stage, sputum should be avoided with the COVID-19 Ag Respi-Strip because false-positive results cannot be excluded with this sample type. Further investigation using fresh nasopharyngeal specimens should clarify why we found one false-positive ICT, whether due to the matrix effect of the viscous sputum or due to *S. aureus* infection. It is worth mentioning that the majority of clinical *S. aureus* specimens (*N* = 9/10) did not give false positive results. An additional limitation in the methods of this study was the absence of a MERS sample for a cross-reactivity study with another member of the betacoronavirus genus. Furthermore, the developed assay has some flaws as it does not differentiate between SARS-CoV-1 and 2.

Sample characteristics may influence the results of the study. According to the manufacturer's IFU, the nasopharyngeal samples must be tested as soon as possible after collection, which concords with the need for rapid diagnosis in this pandemic situation. It should be noted that this clinical validation was performed on leftover sample material after qRT-PCR analysis with a delay of 1 h to 2 days and conservation at 4°C. The delay between sample collection and antigen test processing, as well as the dilution of the sample in the transport media, may have impacted the sensitivity of the assay. As tested with four clinical samples (stored at 2–8°C), the intensity of the reaction slightly diminished after 24 h. Further sample storage studies have to be conducted in the near future. In our population, we did not see any difference between NPS and NPA. Because recently published data report high viral loads in nasopharyngeal, mid-turbinate and nares specimens, we can infer that the ICT could also have an acceptable sensitivity for such specimens. This has previously been described for other viruses and quantitative techniques (20). Evaluations on other swabs, such as foam or polyester swabs, should also be performed.

In regard to our results, the test is sufficiently accurate (with an overall accuracy of 82.6%) for implementation in an integrative diagnostic strategy combining both rapid diagnostic testing based on SARS-CoV-2 antigen detection with the COVID-19 Ag Respi-Strip, molecular POCT and molecular diagnostics on a large automated platform, the latter often requiring a TAT of more than 4 h.

Figure 2 shows that the rapid antigen testing can play a role in patients' arrival at the emergency department in a pandemic context with a high prevalence of COVID-19. Thanks to its specificity of 99.5% and its high PPV of 98.7%, patients with a positive antigen test result could receive immediate care, while antigen test-negative patients will need a CT-scan for triage and the qRT-PCR result for confirmation of SARS-CoV-2. When the peak of the epidemic approaches and prioritization is required, to relieve the emergency department in a situation seeking the implementation of fast hygienic measures and rapid patient care, it is defensible to refrain from confirming the antigen test-positive patients with qRT-PCR.

In the proposal for a diagnostic decision algorithm, the TAT could be contained to 15 min after sample collection for patients with a high viral load (Figure 2). The decision algorithm in Figure 2 shows the potential clinical usefulness of the COVID-19 Ag Respi-Strip for patients suspected of SARS-CoV-2 infection thanks to its high PPV of 98.7%. However, suspected patients suffering from severe comorbidities could benefit from a molecular POCT if available because a higher NPV is desired. But the format and the cost of a molecular POCT limit their use in large screening strategies especially when resources are limited and distribution is delayed.

Since the Hospital Urgency Plan has been declared in Belgium, healthcare workers have been focusing on severely and critically ill COVID-19 patients needing hospitalization. Nasopharyngeal samples for COVID-19-suspected patients are collected upon presentation at the emergency department. These samples possess probably the highest viral load because they are the closest to the date of the onset of symptoms referring to the kinetics of the viral load, as shown in Cao et al. (21).

For this reason, we consider the COVID-19 Ag Respi-Strip to have a relevant place in the diagnostic algorithm at the entry of the emergency department for those COVID-19-suspected patients at risk of developing severe disease who will require hospitalization, according to the definition of a possible COVID-19 case as described by the authorities (Figure 2).

This decision algorithm would prepare all clinical laboratories for the significant increase in the number of specimens that will need to be tested for COVID-19 when large areas of a given country are faced with community transmission. If a 24/7/365 work organization is proposed by the lab, the rapid result for positive cases within 15 min of reception of the specimen would facilitate taking immediate measures to prevent the further spread of the most infectious cases. In a multi-site consolidated clinical microbiology laboratory model, such as the LHUB-ULB (22), a 24 h COVID-19 diagnostic service is provided by dedicated clinical microbiology technologists located in the central lab, while in satellite labs the COVID-19 Ag Respi-Strip is handled 24 h a day by technologists from chemistry or hematology backgrounds after cross-training to competently perform and interpret the results of the rapid test. Samples with a negative ICT result are transferred to the central laboratory for molecular diagnosis.

The post-implementation analysis of the proposed algorithm using samples collected in the LHUB-ULB between the 31st of March and the 7th of April 2020 from patients in four hospitals from Brussels showed 33.3% (325/975) total positive COVID-19 test results, of which 39.7% (129/325) were detected by the COVID-19 Ag Respi-Strip. On epidemic-peak days with a screening capacity of only COVID-19-suspected patients, the proposed algorithm allowed us to avoid 13.2% (129/975) qRT-PCR screening tests, reducing not only expensive qRT-PCR costs but also the consumption of scarce reagents and consumables. Even if the cost of the COVID-19 Ag Respi-Strip test is far lower than the cost of molecular diagnostic methods, the budget impact should be studied within a larger health economic frame. A health economic assessment should question whether a CT-scan would still be needed for triage and taking into account the time gain for the implementation of biosafety measures when an early positive result of a COVID-19 Ag Respi-Strip will help the clinician at the emergency area entry to redirect his/her patient faster, without the need to perform a CT-scan.

Considering risk management and the bottlenecks mentioned in Figure 3, the Ishikawa diagram shows that the COVID-19 Ag Respi-Strip is readily implementable in all clinical laboratories as well as peripheral labs, procuring first-line diagnostic results inside and outside the hospital for GPs and medical specialists all over the country. The development of the COVID-19 Ag Respi-Strip test falls under the immediate priority call from the World Health Organization on the development of POCT. Although the standard operating procedure seems to be written for using the device as a POCT, caution has to be taken before wide application by GPs for three reasons. First, in Belgium, the legal framework has not yet been published for designating the responsibilities of healthcare workers using POCT outside the hospital environment. A proposal for a legal framework has been drafted, closely relating the GP to a clinical laboratory to guarantee traceability within a recognized quality management system and the certification of competent users after training. Second, regarding the biosecurity rules for handling COVID-19-suspicious respiratory samples and the absence of a Biological Safety Cabinet Class II designation at a given GP's office, the GP should possess all necessary protection materials, such as a container for biohazard waste, sterile gloves, disposable gowns, safety goggles (or face shield) and an FFP2 medical mask. Third, in this study, we did not record how many COVID-19 Ag Respi-Strips resulted in weak intensities for the control and test lines. However, in our user-friendly study, the observers mentioned a source of error when reading weakly positive COVID-19 Ag Respi-Strips due to the difficulty in visualizing the color line through the closed tube. When better visualization of the strip—with good light (such as with crystal tubes)—is not available, the lab technician sometimes has to open the test tube in the laminar air flow cabinet and remove the strip with forceps. This type of operation would never be acceptable in a GP environment. At this point, the design of the device is a strip within a closed reaction tube, but R&D will continue to improve its maturity with the perspective of a closed cassette including closed sample handler-enhancing visualization of the test line and a reduction of the risk of user error in the interpretation of the results. Then, a pilot study should evaluate the applicability of the COVID-19 Ag Respi-Strip as a POCT device at GP offices to report the performances obtained in a relevant environment with the intended users.

A reflection on the results leads us to epidemiological questions.

This study was performed during the ascendant phase of the epidemiological curve in Belgium. The results as well as the data interpretation may have resulted differently had the study been performed during another phase of the epidemic. In a low prevalence setting with lower viral shedding, the users of COVID-19 Ag Respi-Strip might experience lower test performance.

For the future use of this ICT, some perspectives are shared here.

At this point, no outpatient population has been sampled due to the lack of material for diagnostic testing. Further studies are needed to test the COVID-19 Ag Respi-Strip in the outpatient population, with special attention paid to healthcare workers on the frontline, such as GPs and pharmacists, who are in regular contact with mildly symptomatic and paucisymptomatic patients. One could consider that in conjunction with public health authorities, isolation measures could eventually be focused on those outpatient individuals with a positive COVID-19 Ag Respi-Strip result. Especially in a subpopulation with a high pre-test prevalence, this could become a tool for managing lock-down situations. The implementation of this measure would depend on the precipitating pandemic situation and the possibility to start supplemental prospective outpatient studies. The study population would depend on the production capacities of this new method, the available resources and the highest public health impact for reducing the transmission.

A major usefulness of the COVID-19 Ag Respi-Strip test would be in the low- and middle-income countries, where molecular assays are available in very few laboratories, mainly only in capital cities. The detection of viral infections in patients attending primary care centres would allow healthcare workers to rapidly identify new outbreak foci and define quarantine measures for high viral shedders and/or suspect patients to limit the spread of the epidemic. This will require the wide distribution of the assay in all care centres, the availability of ancillary material (masks and other PPE, sample collection flocked swabs) and training of the healthcare workers. Although the implementation of COVID-19 Ag Respi-Strip has advantages in a triage scenario (short time-to-result, cost-saving), the sensitivity of the COVID-19 Ag Respi-Strip is too low to meet the requirements of a frontline stand-alone triage test and this limitation must be clearly communicated.

## Conclusion

Our study is the first report evaluating the diagnostic efficacy and operational utility of a disposable rapid antigen test to detect SARS-CoV-2 in nasopharyngeal clinical specimens, which expansively spreads worldwide. Like many of the previously developed lateral flow assays for the detection of viruses in clinical specimens, the COVID-19 Ag Respi-Strip test has lower diagnostic sensitivity compared to currently used gold standard molecular assays such as qRT-PCR. Because it can be performed in 15 min and has a very good specificity of 99.5%, COVID-19 Ag Respi-Strip represents a promising tool for first-line diagnosis of COVID-19 during the ascendant phase of the epidemiological curve. This could accelerate the care process to those patients found positive with the rapid antigen test and limit qRT-PCR analysis. In summary, the role of the COVID-19 Ag Respi-Strip is complementary to the currently used molecular techniques.

## Data Availability Statement

The raw data supporting the conclusions of this article will be made available by the authors, without undue reservation.

## Ethics Statement

The study was approved by the ethical boards (P2020/191 for Hôpital Erasme, CE2020/65 for CHU Brugmann, AK/10-06-41/3907 for CHU Saint-Pierre, S63896 for UZ Leuven). For CHU of Liege, no specific approval was requested to the EC as a leaflet including the following statement is given to all admitted patients: According to the law of the 19th December 2008, any left-over of biological material collected from patients for their standard medical management and normally destroyed when all diagnostic analysis have been performed, can be used for validation of methods. The law authorizes such use except if the patient expressed an opposition when still alive (presume consent). Written informed consent for participation was not required for this study in accordance with the national legislation and the institutional requirements.

## Author Contributions

PM, CJ, AM, VM, DMi, HM, JB, and TL conceptualized the ICT. ND, DMa, LC, SV, PM, KS, NY, SD, KL, GS, MW, MV, and OV did the analytical and clinical validations. PM, ND, and DMa contributed to literature review and the writing of the initial draft. LC, PM, KS, NY, KL, MW, ND, and OV contributed to manuscript revision, data compilation, and figure presentation. All co-authors provided critical review and commentary. OV, ND, and MV contributed to study design, manuscript preparation, literature review, revision, and project administration.

### Conflict of Interest

Two parties have merged their expertise within this article with the intention of delivering a publication in the interest of the public health system: On the one hand, the IVD medical device has been developed by the investigator PM, HM, and JB working for Coris BioConcept and therefore considered a scientific investigators with potential conflict of interest even they don't have any share in this company. TL was also involved in the development of this test and as CEO of Coris Bioconcept he has a potential conflict of interest. On the other hand, the validation as described in the analytical performance study, the clinical performance study and the risk management have been done completely independent from Coris BioConcept, without any conflict of financial interest. All scientific investigators that are external to Coris BioConcept declare having no conflict of interest. Coris BioConcept has offered the reagents for validation and after CE-certification, some kits for diagnosing the homeless people suspected with COVID-19 in Brussels. The remaining authors declare that the research was conducted in the absence of any commercial or financial relationships that could be construed as a potential conflict of interest.
